# Supporting Vulnerable People During Challenging Transitions: A Systematic Review of Critical Time Intervention

**DOI:** 10.1007/s10488-022-01224-z

**Published:** 2022-10-14

**Authors:** Jennifer I. Manuel, Megan Nizza, Daniel B. Herman, Sarah Conover, Laura Esquivel, Yeqing Yuan, Ezra Susser

**Affiliations:** 1 School of Social Work, University of Connecticut, Hartford, CT USA; 2 School of Social Work, Boston University, Boston, MA USA; 3 Silberman School of Social Work, Hunter College, New York, NY USA; 4 Silver School of Social Work, New York University, New York, NY USA; 5 Center for Vulnerable Populations, University of California, San Francisco, CA USA; 6 Mailman School of Public Health, Columbia University, New York, NY USA; 7 New York State Psychiatric Institute, New York, NY USA

**Keywords:** Homelessness, Challenging transitions, Intervention research, Systematic review

## Abstract

Critical Time Intervention (CTI) is designed to reduce the risk of homelessness and other adverse outcomes by providing support to individuals during challenging life course transitions. While several narrative reviews suggest the benefit of CTI, the evidence on the model’s effectiveness has not been systematically reviewed. This article systematically reviews studies of CTI applied to a variety of populations and transition types. Following the Preferred Reporting Items for Systematic Review and Meta-Analysis for protocols (PRISMA-P) guidelines, we reviewed 13 eligible experimental and quasi-experimental studies. Findings were summarized by individual outcome domains, including housing, service engagement use, hospitalization or emergency services, mental health, substance use, family and social support, and quality of life. CTI had a consistent positive impact on two primary outcomes—reduced homelessness and increased service engagement use—among different populations and contexts. Despite the effectiveness of CTI, the specific mechanisms of the model’s positive impacts remain unclear. Implications for practice, policy and research are addressed.

**Supplementary Information:**

The online version contains supplementary material available at 10.1007/s10488-022-01224-z.

## Introduction

Critical Time Intervention (CTI) is a time-limited individual-level intervention designed to reduce the risk of homelessness and other adverse outcomes by providing support to individuals during challenging life course transitions. The intervention is intended to help people make optimal use of existing and new supports, including formal service providers, family, and friends, and provide practical and emotional support during the early phases of the transition process. An important part of CTI is the worker establishing a preliminary relationship and conducting a needs assessment with individuals prior to transition. While the initial CTI studies focused on individuals with serious mental illness and chronic homelessness transitioning from shelters (Susser et al., 1997) and psychiatric inpatient care (Herman et al., 2011), the model has since been adapted for a range of populations and transition types (Herman, 2014; Herman et al., 2007).

CTI emerged in the mid 1980s when New York City and other large US cities were confronting a dramatic growth in the number of homeless persons, including many single adults with severe mental illnesses and substance misuse problems (Valencia et al., 1996). The model’s foundation is based on elements utilized in other evidenced-based models that address the needs of adults with severe mental illness, including small caseloads, assertive outreach, in vivo services, and individualized case management plans. An important distinction between CTI and other assertive outreach models/strategies is that, rather than providing ongoing treatment and direct support, it is a time-limited intervention that aims to root clients within existing systems of community-based services and social support.

CTI has traditionally been delivered in three phases: Initiate Linkages; Try Out; and Final Transfer of Support. An Early Engagement phase occurs prior to transition and involves building a therapeutic relationship with individuals and developing a transition needs assessment. The most intensive phase, Initiate Linkages, begins following discharge and focuses on developing a transition plan, providing intensive support, and identifying the formal and informal resources to whom to transition the care. CTI focuses on a limited range of treatment goals (e.g., housing, mental health, substance use, family and social support, employment) that individuals identify as most important to address for the community integration, thereby personalizing their care strategy to reduce the risk of homelessness and other adverse outcomes. The Try Out phase entails testing and adjusting the formal and informal systems of support that were developed previously. The last phase, Final Transfer of Support, focuses on completing the transfer to a network of formal and informal resources to provide long-term support to the individual. More recently, the importance of the ‘pre-CTI’ phase—the period preceding the actual transition point, when early engagement ideally begins—has been emphasized, leading some descriptions of the model to include four rather than three phases. The CTI phases and activities are described in more detail elsewhere (Center for the Advancement of Critical Time Intervention, 2021, https://www.criticaltime.org/).

Successful implementation of CTI depends on the degree to which key elements of the model are implemented as intended, also known as intervention fidelity. To enhance fidelity, a training program that can be adapted for each context has been developed and is generally used by studies before implementation. Fidelity to CTI is generally measured using the CTI Fidelity Scale, which consists of 15 items that are divided into three sections: client-based, worker-based, and team-based (Conover et al., 2007). The items are measured on a 5-point scale ranging from not implemented to ideally implemented. Fidelity ratings are made of seven core ingredients of CTI: *early assessment and engagement, community-based, phased intervention, decreasing intensity of contacts over time, focused, time-limited and few dropouts*. Ratings are made of two items related to the structure of CTI: *small caseload size and weekly team supervision*. The fidelity scale also includes six items related to the quality of delivering CTI: *worker’s role, supervision, fieldwork coordination, as well as the documentation on the phase plans, progress notes, and closing notes.* The fidelity assessors are aided by worksheets, on which they abstract data and calculate the ratings. The data sources are the documentation by the team members and notes taken by the assessors during observation of team supervision, client records, an interview with the supervisor, and a focus group with the workers.

Despite several narrative reviews describing the impact of CTI (Herman, 2014; Herman & Mandiberg, 2010; Herman et al., 2007), the evidence to date has yet to be systematically reviewed. Additionally, since the last narrative review, the evidence base for CTI has grown substantially with different populations and transition settings nationally and internationally. The primary goal of this review is to summarize and examine the consistency of findings across the CTI studies and their applicability in a variety of populations and transition types through a systematic review of the existing literature.

## Methods

### Search Strategy

We followed the systematic protocol recommended by Preferred Reporting Items for Systematic Review and Meta-Analysis for protocols (PRISMA-P) (Shamseer et al., 2015). We performed a standardized search of the literature using the following databases for peer-reviewed articles dating from 1990 to August 2020: CINAHL, EMBASE, PubMed, and Cochrane. The following search terms were used to identify studies testing the effectiveness of CTI: critical time intervention, critical time, time limited intervention, transitional support, transitional intervention, and transitional assistance. To identify articles not included in our original search, we reviewed reference lists of studies that met inclusion criteria, reviewed existing relevant systematic reviews and general literature reviews (de Vet et al., 2013; Herman, 2014; Herman & Mandiberg, 2010; Herman et al., 2007; Hopkin et al., 2018; Vigod et al., 2013), searched Google and Google Scholar by using different combinations of the terms, and then retrieved unpublished data and gray literature reports from existing networks.

### Study Selection Criteria

A screening tool was specified in advance. Reports were considered eligible for inclusion if they examined CTI or a modified version of CTI using experimental or quasi-experimental designs. Given the broad application of CTI, we did not specify inclusion criteria with respect to populations, transition types, or outcomes. We included reports regardless of their country of origin that were written in or translated into English. We excluded studies without a comparison group, review articles, editorials, commentaries, theoretical articles, and case reports. In addition, we also excluded studies that described CTI as a secondary or partial focus in multi-component interventions.

### Screening and Data Extraction

After we conducted the search, we exported all identified references into EndNote X7. We removed duplicates both automatically using EndNote’s duplicates removal function and manually. Two raters independently screened titles and abstracts of the retrieved publications, excluding reports that did not match our inclusion criteria, and then independently evaluated the full-text publications to confirm eligibility based on our inclusion criteria. Two raters extracted data for all included studies using a standardized Excel form, and a third rater checked each extraction for accuracy. Information was extracted on study design, sample size, follow up, intervention duration, comparison group type, sample population, transitional setting, fidelity rating, and reported outcomes. We found a high level of heterogeneity among studies on these study characteristics, which led us to conduct a narrative systematic review. We categorized and evaluated all outcome measures that were included in randomized controlled trials and quasi-experimental studies.

### Risk of Bias Assessment

To assess risk of bias within each study, we used the Risk of Bias tool (RoB) in the Cochrane Handbook for Systematic Reviews of Interventions (Higgins & Green, 2011). Before beginning the risk of bias assessment, we consulted the Cochrane Handbook to clarify bias categories and criteria for judgements (Higgins & Green, 2011). A spreadsheet was created that contained a separate, identical table for each study, comprised of a set of categories for each element of potential bias: Selection Bias (Random Sequence Generation), Selection Bias (Allocation Concealment), Performance Bias (Blinding—participants & personnel), Detection Bias (Blinding—outcome assessment), Attrition Bias (Incomplete Outcome Data), Reporting Bias (Selective Reporting). We did not assess for Other Bias (Other Sources of Bias), though we acknowledge the possibility of other sources of bias for some studies. Based on the Cochrane Handbook, descriptions of each bias category and rating anchors were provided to each rater. Notes, including direct quotes from studies and rater comments and justifications, were documented along with each rater’s independent assessment.

To establish consistency and minimize the impact of rater subjectivity, we used an iterative process involving independent reviews by multiple raters. Three raters conducted the risk of bias review (JIM, MN, DB). During the first round of reviews, each rater reviewed the same three studies independently then went through each rating of each study together. If ratings did not align, raters gave justifications and again reexamined the study together until consensus was reached. The second round of reviews included another three studies with the same process. The third review consisted of two raters (JIM, MN) assessing the remainder of the studies independently. After completing the independent ratings, all three raters met to discuss discrepancies in ratings of each study until consensus was achieved for all studies. If raters identified a need for more information, study authors were contacted. If a study was rated high or unclear on at least two bias domains, they were given an overall rating of high risk of bias.

## Results

### Search Results

**Fig. 1:**
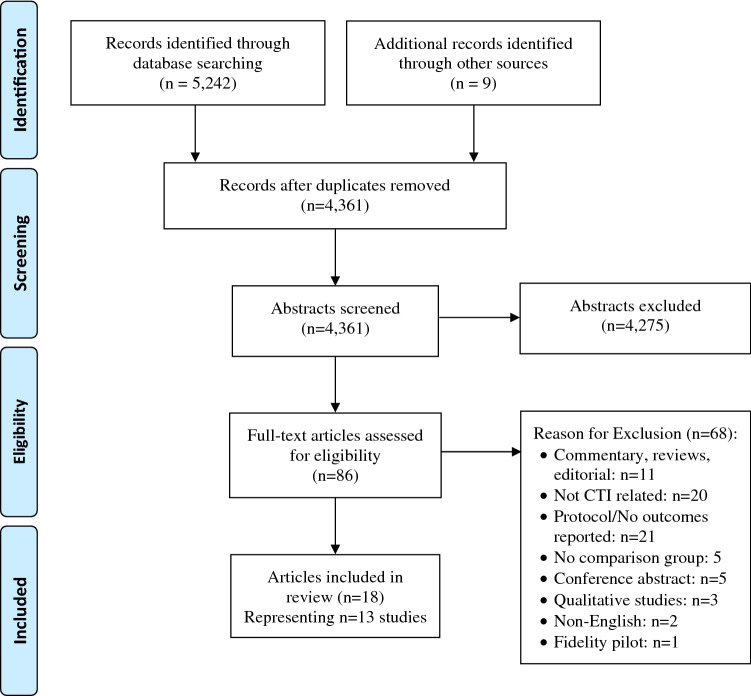
Preferred reporting items for systematic reviews

The results of the systematic search and selection process are summarized in Fig. 1. The search identified 5242 citations from database searching and nine citations were identified through other sources. Of these, 890 citations were duplicates. A total of 4361 abstracts were screened and reviewed, and 4275 ineligible studies were excluded. The full text of the remaining 86 articles was assessed further for inclusion. A total of 68 full-text articles were excluded. We excluded articles that were non-English; classified as conference abstracts, commentary, reviews, protocols, fidelity pilots or qualitative studies; or described transition interventions where CTI was not a central component of the intervention. A total of 18 articles were included in the review, representing data from 13 original studies. One of the included studies was from the grey literature, which includes unpublished reports, working papers, government documents, and evaluations.

### Characteristics of Included Studies

Table 1 describes the characteristics of the included studies. Of the 13 original studies, 10 were randomized controlled trials and three were quasi-experimental studies. The majority of studies were conducted in the United States (n = 8), one in Brazil, two in the Netherlands, one in the United Kingdom, and one in Canada. Sample sizes ranged from 71 to 484 participants and follow-up periods ranged from 3 to 24 months. Ten studies reported attrition at follow-up, ranging from 2.7 to 44%. Nine studies discussed fidelity monitoring, of which 4 studies reported an overall fidelity score of 3 out of 5 or better (indicating at least fair model fidelity). Two studies used a modified fidelity scale that included 12 items representing compliance fidelity, or the degree to which the key elements of CTI were implemented (8 items) and the extent to which the intervention is well-documented (4 items) (de Vet et al., 2017; Lako et al., 2018).

**Table 1:** Description of critical time intervention studies by study design

Author	Participants	Transition setting	Intervention groups	Intervention duration	Follow-up period	Attrition	Overall fidelity score
*Experimental*							
Crampton et al. (2020), Collins et al. (2020)	*N* = 273 homeless families with children	Homeless shelter in Cuyahoga County, Ohio	CTI (*n* = 135) vs. treatment as usual (*n* = 138)	12 months post enrollment	12 and 24 months	Not reported	Not reported
Silva et al. (2017)	*N* = 71 adults with serious mental illness	Psychiatric hospital in Rio de Janeiro, Brazil	(CTI: *n* = *35*; treatment as usual: *n* = 38)	9 months post discharge	9 months	2.7%	Fidelity monitoring revealed that CTI was implemented as intended. Fidelity score not reported
de Vet et al. (2017)	*N* = 183 homeless adults	18 shelters in the Netherlands	CTI (*n* = 94) vs. treatment as usual (*n* = 89)	9 months post discharge	9 months	5%	Using an adapted, brief fidelity scale on a random sample of 35 participants, an average score of 3 (fairly implemented) on a 1–5 scale was achieved; a score of 3 or higher was achieved on 8 of the 12 fidelity items
Dixon et al. (2009)	*N* = 135 veterans with serious mental illness	Four acute inpatient psychiatric hospitals in Baltimore, Maryland	CTI (*n* = 64) vs. treatment as usual (*n* = 71)	3 months post discharge	6 months	14%	80% and 63% of CTI participants received scores of 3 and 4, respectively, on a scale from 1 (not at all) to 5 (very much so)
Herman et al. (2011), Tomita and Herman (2012, 2015), Tomita et al. (2014)	*N* = 150 formerly homeless individuals with severe mental illness	Two inpatient psychiatric hospitals in New York, New York	CTI (*n* = 77) vs. treatment as usual (*n* = 73)	9 months post discharge	18 months	22%	Fidelity monitoring revealed that CTI was implemented as intended with some variation in pre-discharge contacts. Fidelity score not reported
Lako et al. (2018)	*N* = 136 homeless women with history of intimate partner violence	Nine shelters for women who experienced intimate partner violence in the Netherlands	CTI (*n* = 70) vs. treatment as usual (*n* = 66)	9 months post-discharge	9 months	6%	Average score of 3 (fairly implemented) on scale of 1–5 was achieved
Samuels et al. (2015), Shinn et al. (2015)	*N* = 210 homeless mothers with a mental disorder and/or substance use disorder prior to shelter admission	Four family homeless shelters in New York, New York	CTI (*n* = 97) vs. treatment as usual (*n* = 113)	9 months post discharge	15 months	27%	Fidelity assessment revealed that the implementation of CTI was implemented as intended. Fidelity score not reported
Shaw et al. (2017)	*N* = 150 men with serious mental illness	Eight prisons in the United Kingdom	CTI (*n* = 72) vs. treatment as usual (*n* = 78)	6 weeks post release	6 weeks, and 6 and 12 months	32%	Average score of 4.04 on a scale of 1–5 was achieved; the range of average scores per subscale is as follows: Components: 2.87–5.00 Structure: 5.00 Quality: 2.87–5.00
Susser et al. (1997), Herman et al. (2000)	*N* = 96 homeless men with serious mental illness	Homeless shelter in New York, New York	CTI (*n* = 48) vs. treatment as usual (*n* = 48)	9 months post discharge	18 months	2%	Adherence to CTI protocol monitored. Fidelity score not reported
Stergiopoulos et al. (2017)	*N* = 166 frequent users (five or more visits) of an emergency department, with at least one visit for a mental health or substance use reason	Emergency department in Toronto, Canada	CTI (*n* = 83) vs. treatment as usual (*n* = 83)	4–6 months post discharge	12 months	9%	Not reported
*Quasi-experimental*							
Kasprow and Rosenheck (2007)	*N* = 484 former or at-risk homeless veterans with severe mental illness	Eight inpatient psychiatric hospitals in the United States	CTI (*n* = 206) vs. treatment as usual (*n* = 278)	6 months post discharge	12 months	44%	Not reported
Nossel et al. (2016)	*N* = 97 frequent users of psychiatric emergency services (3 or more emergency room visits in prior year)	Psychiatric emergency room in New York, New York	CTI (*n* = 47) vs. treatment as usual (*n* = 50)	6 months post discharge	12 months	Not reported	Not reported
Shaffer et al. (2015)	*N* = 373 individuals with serious mental illness or co-occurring mental health and substance use disorder	Six inpatient psychiatric hospitals in western Pennsylvania	CTI (*n* = 149) vs. treatment as usual (*n* = 224)	3 months post discharge	3 months	Not reported	Most CTI activities were implemented at a high rate across the three phases. Fidelity score not reported

Included studies represented a range of populations and transition types, including individuals with serious mental illness and chronic homelessness, individuals with co-occurring mental health and substance use disorders, veterans, individuals who were being released from prison or jail, homeless families, homeless women, and women with history of intimate partner violence. CTI was implemented for a variety of transitions, including from homeless shelters, transitional living communities, inpatient psychiatric hospitals, emergency room, and prisons or jails. Most studies also involved a shortened intervention period from the original 9 months to 3 to 6 months in duration. Outcomes also varied widely between studies, with housing and service engagement as most prominent. Other outcomes examined were hospitalization or emergency services, mental health, substance use, family and social support, and quality of life. Most often, different instruments and operationalizations of outcomes were used across the studies (see Online Appendix A). It is important to note that the designation of outcomes as primary or secondary varied and depended on the study goals and population or transition setting. As such, we report all outcomes by domain without such designation in the results.

### Methodological Risk of Bias Assessment

Details of the risk of bias assessment are provided in Table 2. All 10 experimental studies were rated as exhibiting low risk of bias and all three quasi-experimental studies received ratings of high risk of bias. For Selection Bias domains (Random Sequence Generation and Allocation Concealment), even if procedures were not described in great detail, if groups were found to be comparable at baseline, a low bias rating was given. Of note, Silva et al. (2017) had a relatively small sample size, which is a limitation, and though the study appeared to follow appropriate, unbiased procedures, it ended up with unbalanced groups with respect to gender. Nevertheless, Silva et al. (2017) was still rated as low risk. The publications with a high-risk rating were primarily limited methodologically due to absence of randomization and high risk of selection bias. Performance Blinding, wherein both participants and personnel were blind to knowledge of which intervention the participant received, was impossible in nearly all studies due to the nature of the intervention. According to Cochrane guidelines, if raters acknowledge no blinding or incomplete blinding, but do not believe that such would have affected the outcome, then a low rating is appropriate as long as the judgment is justified (Higgins & Green, 2011). The same rating logic applies to Detection Bias when outcome assessors have knowledge of the participants’ intervention status; if the raters believe that lack of blinding to the intervention that the participant received by outcome assessors has not influenced the outcome measurement, then a low rating is appropriate as long as the judgment is justified (Higgins & Green, 2011). Study attrition levels were relatively low with only three studies reporting more than 25% attrition at follow up. Crampton et al. (2020) had a shortage of specific details regarding missing data, which accounts for our only unclear rating.

**Table 2:** Assessment of bias

Author	Selection bias Random sequence generation	Selection bias Allocation concealment	Performance blinding Blinding—participants & personnel	Detection bias Blinding—outcome assessment	Attrition bias Incomplete outcome data	Reporting bias Selective reporting	Score
*Experimental*							
Crampton et al. (2020) Collins et al. (2020)	Low	Unclear	Low	Low	Low	Low	Low
Silva et al. (2017)	Low	Low	Low	Low	Low	Low	Low
de Vet et al. (2017)	Low	Low	Low	Low	Low	Low	Low
Dixon et al. (2009)	Low	Low	Low	Low	Low	Low	Low
Herman et al. (2011), Tomita and Herman, (2012, 2015), Tomita et al. (2014)	Low	Low	Low	Low	Low	Low	Low
Lako et al. (2018)	Low	Low	Low	Low	Low	Low	Low
Samuels et al. (2015), Shinn et al. (2015)	Low	Low	Low	Low	Low	Low	Low
Shaw et al. (2017)	Low	Low	Low	Low	Low	Low	Low
Susser, et al. (1997), Herman et al. 2000)	Low	Low	Low	Low	Low	Low	Low
Stergiopoulos et al. (2017)	Low	Low	Low	Low	Low	Low	Low
*Quasi-experimental*							
Kasprow and Rosenheck (2007)	High	High	High	High	Low	Low	High
Nossel et al. (2016)	High	High	Low	Low	Low	Low	High
Shaffer et al. (2015)	High	High	Low	Low	Low	Low	High

### Efficacy of CTI

Table 3 presents the overall impact in each outcome domain for each study, indicating when two or more measures of an outcome were reported. We summarize each outcome domain below, highlighting results from experimental and quasi-experimental studies.

**Table 3:** Summary of findings from critical time intervention studies

Author	Housing	Service engagement	Hospitalization/emergency services	Mental health	Substance use	Family support	Social support	Quality of life
*Experimental studies*								
Crampton et al. (2020), Collins et al. (2020)	Positive_4_							
Silva et al. (2017)				No effect		No effect	No effect	
de Vet et al. (2017)	No effect			Positive^a^	No effect_2_	Positive	No effect	No effect
Dixon et al. (2009)		Positive_4_	Positive_2_	No effect_3_		No effect	Positive	Mixed_6_
Herman et al. (2011) Tomita and Herman (2012, 2015), Tomita et al. (2014)	Positive_2_	Positive_5_	Positive_1_			Positive_2_		
Lako et al. (2018)				Mixed_4_		No effect	No effect	No effect
Samuels et al. (2015), Shinn et al. (2015)	Positive	Positive^b^		Mixed_6_				
Shaw et al. (2017)		Positive_2_						
Susser, et al. (1997), Herman et al. (2000)	Positive_5_			Mixed_3_				
Stergiopoulos et al. (2017)		No effect_1_	No effect_3_	No effect	No effect			Mixed_3_
*Quasi-experimental studies*								
Kasprow and Rosenheck (2007)	Positive_3_			Positive	Positive_4_			
Nossel et al. (2016)		Positive^b^	No effect					
Shaffer et al. (2015)		No effect	Mixed_4_					

Subscripts were used when multiple outcomes were reported within each domain. For multiple outcomes with inconsistent effects, we indicated findings as positive when the majority (> 70%) reported a positive direction and inconsistent otherwise (de Vet et al., 2013). Reported positive effects were significant at *p* ≤ .05 except when noted otherwise

*Positive* CTI had a positive effect, *Negative* CTI had a negative effect on outcomes, *No effect* No group difference in outcomes, *Mixed* inconsistent effects

^a^Significant at *p* ≤ 0.10

^b^No significance tests reported

#### Housing

Housing (versus homelessness) is one of the most prominent and consistent outcomes studied. Of the six experimental and quasi-experimental studies examining the impact of CTI on housing outcomes, five showed positive effects. Of these five, four were experimental studies (Collins et al., 2020; Herman et al., 2011; Samuels et al., 2015; Susser et al., 1997) and one quasi-experimental (Kasprow & Rosenheck, 2007). All five studies involved transitions from homeless shelters (Collins et al., 2020; Samuels et al., 2015; Susser et al., 1997) and psychiatric hospitals (Herman et al., 2011; Kasprow & Rosenheck, 2007).

The first randomized trial of CTI versus usual services involved 96 homeless men with a serious mental illness leaving the shelter (Susser et al., 1997). CTI was associated with a three-fold reduction in the risk of homelessness over 18 months following discharge, a finding that was sustained following the 9-month intervention; significantly fewer homeless nights among CTI participants; and a significant reduction was found in extended homelessness (more than 54 nights), the most serious form of homelessness. In the second randomized trial, Herman et al. (2011) compared CTI versus usual services among individuals with serious mental illness and chronic homelessness following psychiatric hospital discharge and found a five-fold reduction in the prevalence of homelessness among participants assigned to CTI compared to those assigned to usual services. The risk of homelessness was reduced even further (by tenfold) for CTI participants receiving three or more predischarge contacts with their CTI worker, a finding that supported the value of the early engagement phase in CTI. Like Susser et al. (1997), this study also reported enduring impacts of CTI on homelessness risk during the 9 months following the end of the intervention (Herman et al., 2011). In a randomized trial of homeless families, Family-CTI led to a significant likelihood of being rehoused more quickly compared to usual services during the study period (Samuels et al., 2015). Based on an interim analysis of another randomized study involving families, Collins et al. (2020) reported lower emergency shelter use among CTI participants compared to control participants at 24 months post enrollment.

In a quasi-experimental study of CTI versus usual services among homeless veterans, Kasprow and Rosenheck (2007) found largely positive results on housing outcomes over 12 months. CTI participants were significantly more likely to spend more days housed (i.e., living in own home or with others) than control participants and significantly fewer days in institutional settings (i.e., hospital, residential treatment, or jail). However, there were no significant group differences in prevalence of homelessness, but there were significant reductions in the number of homeless nights for both study groups during the follow-up period (Kasprow & Rosenheck, 2007).

One randomized study from Europe reported no significant differences in housing outcomes between CTI and treatment-as-usual (de Vet et al., 2017), however this finding may be partially explained by implementation challenges. In this study, which enrolled homeless adults staying at a shelter for less than 14 months and moving to independent housing, recurrent homelessness was infrequent in both conditions (de Vet et al., 2017). In addition, treatment as usual in the Netherlands, this study’s location, was highly intensive compared to usual care in the United States (de Vet et al., 2017) and may not have differed sufficiently from CTI to detect program impacts.

#### Service Engagement

Of the seven studies that examined service engagement, four experimental studies reported an overall positive impact on service engagement following CTI (Dixon et al., 2009; Nossel et al., 2016; Samuels et al., 2015; Shaw et al., 2017). One study investigating service engagement (inclusive of CTI engagement) as a process variable reported favorable findings (Tomita & Herman, 2015).

In a randomized study of a brief 3-month model (Brief CTI) with veterans following inpatient psychiatric hospitalization, results suggested significantly greater continuity of care in post-discharge mental health and substance use services compared with usual discharge planning (Dixon et al., 2009). Nossel and colleagues (2016) found a significant increase in outpatient services in the CTI group versus comparison group. In the Family-CTI study, participants randomized to CTI were connected to more mental health services than those in the control group (Samuels et al., 2015). Shaw et al. (2017) reported significant improvement in post-release continuity of mental health care, including use of care coordinators and psychiatrists, among men with serious mental illness transitioning from prison who were randomized to CTI versus treatment as usual.

Two studies found mixed results in the impact of CTI on service engagement. Shaffer and colleagues reported no significant group differences in linkage to mental health- and substance use-related service visits during this period.

Tomita and Herman (2015) found improved continuity of care (i.e., perceived access to care and fewer changes in case managers) were observed in the CTI group compared to the usual care group. The authors also found improved continuity of care, inclusive of CTI engagement, was significantly associated with a reduction in psychiatric rehospitalization and homelessness, suggesting the potential mediating role of continuity of care (Tomita & Herman, 2015).

#### Hospitalization or Emergency Services

Five studies examined hospitalization or emergency services, of which two reported findings in favor of CTI (Stergiopoulos et al., 2017; Tomita & Herman, 2012). Tomita and Herman (2012) found that participants assigned to CTI experienced significantly fewer psychiatric rehospitalization days during the 9 months following the end of the intervention, compared to those assigned to usual care. Results from a randomized trial involving frequent users of an emergency room services indicated a reduction in emergency room visits but this decrease was not significant (Stergiopoulos et al., 2017).

No significant group differences were found in post-discharge hospitalization and emergency room visit outcomes in a randomized study of a Brief CTI model with veterans (Dixon et al., 2009). In their quasi-experimental study of Brief CTI (3 months), Shaffer and colleagues reported a lower early psychiatric readmission rate within 30 days of discharge among participants in the CTI group versus comparison group, but the groups did not differ significantly with respect to long-term readmission rates (31–180 days). Nossel et al. (2016) found no significant group differences in psychiatric emergency or inpatient services, although both groups had a significant reduction in these services in the 6 months after the index emergency room visit. The authors attribute the latter non-significant findings to a regression to the mean effect.

#### Mental Health

One experimental study (de Vet et al., 2017) and one quasi-experimental studies (Kasprow & Rosenheck, 2007) reported positive results. In their 9-month randomized trial of CTI versus treatment as usual involving homeless individuals transitioning from shelters, de Vet et al. (2017) found a significant difference in psychological distress in favor of CTI participants but only among those experiencing less social support from other sources. Kasprow and Rosenheck (2007) reported significantly fewer mental health problems at the 3-, 6-, and 9-month follow-up intervals for CTI versus usual care participants, although both groups showed significant declines in mental health problems over the 1-year follow up.

Three experimental studies reported mixed results in the impact of CTI on mental health outcomes. Lako et al. (2018) reported significantly fewer post-traumatic stress disorder symptoms in the CTI group compared to those assigned to usual care. However, no significant differences in other mental health outcomes (i.e., symptoms of depression, psychological distress) were observed. The randomized trial of Family-CTI reported significant reductions in mental health symptoms among preschool-aged children (1.5–5 years) and adolescents aged 11–16 years assigned to the experimental group (Shinn et al., 2015). However, no significant group differences were observed in mental health outcomes among homeless mothers (Samuels et al., 2015). Herman et al. (2000) found a significant reduction in negative symptoms of psychosis among participants in the CTI versus usual care groups, a finding which the authors suggest might be partially explained by the increases in social support and an enhanced positive therapeutic relationship between clients and providers. However, no significant group differences were found in positive and general symptoms of psychosis.

Three experimental studies showed no significant impact of CTI on mental health outcomes (Silva et al., 2017; Dixon et al., 2009; Stergiopoulos et al., 2017).

#### Substance Use

Three studies examined substance use as an outcome, and only one quasi-experimental study reported positive results. Kasprow and Rosenheck (2007) reported significantly lower alcohol and drug use severity scores as measured by the Addiction Severity Index (McLellan et al., 1992) among homeless veterans receiving CTI versus those in usual care.

Two studies, both experimental studies, found no significant impact of CTI on substance use outcomes (de Vet et al., 2017; Stergiopoulos et al., 2017).

#### Family and Social Support

Four studies examined family and social support outcomes (Silva et al., 2017; de Vet et al., 2017; Dixon et al., 2009; Lako et al., 2018) and one study examined family support as a mediator, or mechanism through which CTI impacted the outcome (Tomita et al., 2014).

Three studies reported favorable outcomes for this domain (de Vet et al., 2017; Dixon et al., 2009; Tomita et al., 2014). de Vet et al. (2017) found that CTI had a significant impact on family support at follow up, however, no significant differences in non-family social support were observed between CTI and usual care. In contrast to these findings, Dixon et al. (2009) found a significant impact of CTI on the frequency of social contacts but not family contacts. An analysis of data from a randomized trial of CTI versus usual care among individuals with serious mental illness and a history of homelessness revealed significant improvements in family contact and the quality of family relationships among CTI participants (Tomita et al., 2014). This study also reported evidence suggesting that improved family relations may be a potential mechanism through which CTI reduced the risk of psychiatric rehospitalization.

Two randomized trials reported no significant impact of CTI versus usual care on family and social support domains (Silva et al., 2017; Lako et al., 2018).

#### Quality of Life

Of the four studies that examined quality of life as an outcome, two produced mixed results and two no positive results. In their randomized trial of Brief CTI with veterans with serious mental illness, Dixon et al. (2009) found participants assigned to Brief CTI had greater satisfaction with legal and safety issues than those assigned to usual care but no significant differences were found in satisfaction with other quality of life indicators (i.e., living situation, daily activities and functioning, finances, work, school, and health). In a randomized trial of frequent users of emergency care services, Stergiopoulos et al. (2017) reported significantly improved disease-specific quality of life in participants receiving CTI versus usual care but not health-related quality of life at 12 months. However, the authors note that this impact on disease-specific quality of life should be considered with caution given that only 50% of participants had complete data at both time points (Stergiopoulos et al., 2017) and a second analysis with a global indicator of quality of life showed no significant differences between groups. Two randomized studies using the Lehman Quality of Life Interview found no significant impact of CTI on general life satisfaction (de Vet et al., 2017; Lako et al., 2018).

## Discussion

### Summary of Findings

This is the first systematic review to our knowledge summarizing the efficacy of CTI across multiple domains. This review extends the earlier narrative reviews of CTI (Herman et al., 2007; Herman & Mandiberg, 2010; Herman, 2014), which reported on fewer randomized controlled trials with different target populations. We found 18 reports based on 13 studies, most involving randomized controlled trials. Like the original CTI trials (Herman et al., 2011; Susser et al., 1997), the majority of studies recruited participants with current or a history of homelessness. Despite the original 9-month intervention timeframe, most studies used abbreviated timeframe ranging from 3 to 6 months. Justifications for an abbreviated model range from maximizing program capacity (Kasprow & Rosenheck, 2007; Nossel et al., 2016) to focusing specifically on continuity of care outcomes (Dixon et al., 2009; Nossel et al., 2016; Shaffer et al., 2015; Shaw et al., 2017; Stergiopoulos et al., 2017). Other adaptations offered CTI alongside with ancillary services and supports, including trauma-informed care (Crampton et al., 2020); the Strengths Model (Lako et al., 2018); and family-based care (Samuels et al., 2015). Nossel et al. (2016) employed peer specialists as CTI workers as a way to increase the acceptability of the intervention to study participants. Eleven studies described the extent to which the interventions were delivered in accordance with model criteria, although only four studies reported an overall fidelity score.

Three key findings emerged from our review. First, there is consistent evidence that CTI has a positive impact on housing and service engagement use outcomes in the United States. These outcome domains were among the most commonly studied. These results align with CTI’s focus of linking individuals to services and supports in the community to address areas of need critical for successful transition. These findings highlight the practical and adaptable nature of CTI in which the model can be implemented successfully across varied settings and populations (Herman, 2014).

Notably, the studies conducted outside of the U.S. revealed inconsistent homelessness and service engagement findings. This inconsistency may be due to cross-country differences or implementation challenges. For example, de Vet et al. (2017) suggest that the lack of impact on housing outcomes they observed in the Netherlands may be because recurrent homelessness was rare in both study conditions. The authors also believed the impact of CTI on housing outcomes might have been stronger had the intervention been delivered with greater model fidelity. Stergiopoulos et al. (2017) suggest that the complex needs of the frequent emergency room users with mental illness or addiction they studied in Canada may not have been adequately addressed by CTI. The authors also noted that the delivery of the intervention may have had a greater impact had it more closely followed the model’s core components and tasks.

Less consistent results were found with respect to other domains (i.e., hospitalization or emergency services, mental health, substance use, family and social support, and quality of life). The inconsistent findings may be a function of the priority areas defined by individuals in collaboration with their CTI case managers. If the intervention work is centered on the priorities of obtaining and retaining housing and service engagement, especially during the early phases of the intervention, then other issues like family and social support and quality of life may receive less attention. Perhaps if studied interventions had focused on engaging more informal resources, they would have had more positive gains in other domains. Furthermore, it may take longer to see the intervention’s impact on these ancillary domains given the chronic nature of homelessness and mental illness prevalent in many sample populations. Finally, these domains are likely more resistant to change using a time-limited care coordination approach, especially in studies using the abbreviated model of CTI (i.e., 3–6 months) applied in six of the studies. Nevertheless, studies with long-term follow-up assessments are needed to assess whether changes in CTI are sustained over time.

While the optimal duration of CTI is unclear, a second key finding of this review is the successful use of the 9-month model with a variety of populations and settings. Results on the implementation of CTI using shorter durations are less clear, suggesting the need for studies to compare the impacts of CTI of varying duration and intensity. Additionally, we know little about for whom the intervention works and under what conditions. For example, research is needed that moves beyond examining the average treatment effects by identifying the intervention effects across subgroups with respect to demographic (i.e., age, gender, race/ethnicity) and clinical (i.e., symptom severity) characteristics, and for whom might varying durations and intensity work best.

Third, like many other empirically supported models, we found limited evidence that addresses how CTI achieves its positive impacts. The specific mechanisms of how CTI leads to reductions in homelessness and other outcomes remain unclear. Indeed, only one of the randomized trials (Tomita et al., 2014) examined potential mediating paths through which CTI may operate. More research is clearly needed to map CTI’s program components onto specific mediators and investigate whether and how these mediators affect specific outcomes.

### Limitations of Included Studies

Findings from this review should be interpreted with caution given the high degree of variability in the samples and methodological design and rigor of the studies reviewed. Furthermore, making comparisons between studies is limited due to a lack of standardization, inconsistent fidelity assessment and reporting, varying lengths of intervention, diversity in samples and outcomes, inconsistency of measures, and variability in methodological design and rigor. Five of the 10 randomized controlled trials were conducted outside the US, including Brazil, Canada, Netherlands, and United Kingdom. While several of these studies are small, making it difficult to detect effects, it is important to note the substantial differences between countries with respect to the strength of the safety net and organization of health care systems that make it challenging to compare results across these studies. The studies also relied heavily on participant self-report data, which may lead to inaccuracies in the reporting of treatment effects. Although most of the outcome measures in the studies reviewed have strong psychometric properties, future studies should consider including real-time measures of behaviors and experiences (i.e., ecological momentary assessments) or more objective measures (e.g., electronic health records) when possible.

### Limitations of the Review

We note several limitations to our review. First, we did not examine the costs of CTI, although one of the included studies found CTI to be cost effective (Jones et al., 2003; described in Online Appendix A). Second, the included studies were implemented with a variety of populations and settings using varying durations, making it difficult to compare across studies. Third, the number of outcomes examined, as well as the lack of consistency in most, precluded a formal meta-analysis. As the body of CTI evidence grows, we hope to conduct such an analysis on selected outcomes. Another limitation is that the bias assessment was conducted for each study overall and did not separately assess the risk of bias for each outcome of each study. Therefore, while a study's overall risk of bias might be rated as low, the risk of bias for different outcomes within each study is not reflected in the overall ratings. The Cochrane assessment scheme excludes key implementation processes (e.g., program specification, training and supervision, fidelity, intervention dose/attrition) that also may impact the quality of studies.

### Implications for Future Research

CTI showed a fairly consistent positive impact on homelessness and increased service engagement across diverse populations, suggesting the versatility of CTI for different populations and transitions. To properly inform policymakers and practitioners, future studies should be carefully designed with an aim for greater consistency in outcomes evaluated and enhanced standardization of measurements. Additionally, several ancillary, longer-term outcomes of CTI, including mental health, substance use, family and social support, and quality of life, require greater study. Future studies are needed to examine structural adaptations to the model, such as the intervention duration and delivery approaches (e.g., the use of peer specialists). Future adaptations of CTI should be guided by implementation science to ensure contextual factors are considered when tailoring the model to a specific population or transition setting. Additionally, we need greater attention to how these models will be sustained, especially in fragmented systems of care like that of the U.S.

Another important implication of our review is the need to predict which model components facilitate favorable outcomes. Formal testing of mediating and process variables need to be incorporated into future research. More research is also needed to understand subgroup effects and the specific adaptations needed to better serve those for whom the current model is less effective. The articles we reviewed contained limited information on important implementation processes, including training, supervision, and fidelity assessment processes. More consistent use of the CTI fidelity scale in future research is critical for exploration of key components of the model. As CTI continues to be broadly disseminated into routine practice settings, it would also be fruitful to study the cost-effectiveness of intervention duration and delivery approaches, as well as the challenges and strategies in its uptake.

## Conclusion

Mitigating the challenges of community reintegration and coordinating continuing care during service transitions is essential for persons with mental health, housing, and other needs. CTI can serve as a bridge during periods of transition when existing service systems are unable to provide the level of support ideally needed. Future research is needed to address unanswered questions about this promising model so that policymakers and practitioners can make maximum use of it to reduce the risk of homelessness and other adverse outcomes among vulnerable individuals served by a variety of healthcare and social service delivery systems.

## Supplementary Information

Below is the link to the electronic supplementary material.

Supplementary file1 (PDF 108 kb)
