# Supplementary material for: Supporting Vulnerable People During Challenging Transitions: A Systematic Review of Critical Time Intervention
Source: Adm Policy Ment Health. 2022 Oct 14;50(1):100–13. doi: 10.1007/s10488-022-01224-z (PMC9832072; doi:10.1007/s10488-022-01224-z)
Supplement: Supplementary file 1 — Supplementary file1 (PDF 108 kb) [file 10488_2022_1224_MOESM1_ESM.pdf]

Appendix A. Summary of reported client-level outcomes

Author	Follow-up	Outcome	Instrument	Effect
**Experimental**				
Crampton et al 2020 (Collins et al 2020)	24 months	Housing: use of emergency shelter	Homeless Management Information System	χ^2 = 6.20, p<.05
		Housing: use of coordinated assessment	HMIS	χ^2 = 5.88, p<.05
		Housing: use of rapid rehousing	HMIS	No effect
		Housing: use of any homeless services (emergency shelter, coordinated assessment, rapid rehousing)	HMIS	χ^2 = 5.67, p<.05
da Silva et al 2017	9 months	Mental Health: mean score of the mental health component subscale	SF-12	No effect
		Family Support: mean score of personal and social relationships subscale	Personal and Social Performance (PSP)	No effect
		Social Support: mean score of personal and social relationships subscale	PSP	No effect
de Vet et al 2017	9 months	Housing: number of days rehoused, defined as living in conventional indepednent housing or accommodation permanently provided by relatives, friends, or acquaintances	Residential Follow-Back Calendar	No effect
		Mental Health: mean global severity index score	Brief Symptom Inventory	Mean difference: -.14, 95% CI: -.29-.01, p<.10
		Substance Use: excessive alcohol use (i.e., five or more drinks a day) in the past 30 days	European Addiction Severity Index	No effect
		Substance Use: cannabis use in the past 30 days	European Addiction Severity Index	No effect
		Family Support: mean score of how often relatives are available to provide practical and emotional support	5-item scale from the RAND Course of	OR=.36, 95% CI: .02-.71
		Social Support: mean score of how often friends or acquaintances are available to provide practical and emotional support	5-item scale from the RAND Course of	No effect
		Quality of Life: two-item average quality of life score	Lehman's Brief Quality of Life Interview	No effect
Dixon et al., 2009	6 months	Service Engagement: number of days to first mental health or substance use outpatient visit since hosptial discharge	VA performance measures	Hazard Ratio: 2.73, 95% CI: 1.80-4.15
		Service Engagement: percentage of participants who had a greater number of mental health and substance use visits	VA performance measures	All patients in B-CTI group had an outpatient visit, thus no response
		Service Engagement: number of mental health or substance use outpatient treatment visits	VA performance measures	Event Rate Ratio: 2.14, 95% CI: 1.58-2.91
		Service Engagement: greater continuity of care defined as a greater number of two-month blocks with two or more outpatient visits	VA performance measures	Mean difference: .63, 95% CI: .28-.97
		Hospitalization/Emergency Services: rate of inpatient hospital days	VA performance measures	Event Rate Ratio: 1.28, 95% CI: .75-2.17
		Hospitalization/Emergency Services: rate of emergency room visits	VA performance measures	Event Rate Ratio: .67, 95% CI: .32-1.36
		Mental Health: total mental health severity score	Brief Pscyhiatric Rating Scale	No effect
		Mental Health: total positive symptoms subscales	Brief Pscyhiatric Rating Scale	No effect
		Mental Health: toatl negative symptoms subscale	Brief Pscyhiatric Rating Scale	No effect
		Family Support: mean score of family relations subscale	Lehman Quality of Life Interview	No effect
		Social Support: mean score of social relations subscale	Lehman Quality of Life Interview	p=.013
		Quality of Life: mean scores of eight subscales...	Lehman Quality of Life Interview	
		Living situation		No effect
		Daily activities and functioning		No effect
		Finances		No effect
		Work and school		No effect
		Legal and safety issues		p=.026
		Health		No effect
Herman et al., 2011 (Baumgartner & Herman, 2012; Tomita & Herman, 2012; Tomita, Lukens, & Herman, 2014)	9 and 18 months	Housing: any homelessness	Personal History Form	Last 3 intervals: OR=.22, 95% CI: .06-.88
		Housing: number of homeless nights	Personal History Form	Last 3 intervals: p<.001
		Service Engagement: continuity of care defined as...		
		Mean score of perceived ease of access to care	4-item measure adapted from Bindman and	18 months: b=.73; p=.02
		Stability in patient-provider relationship as measured by any changes in providers (psychiatrist, case manager, and therapist)	Participant Interview	Case manager/therapist at 9 months: χ^2 = 4.0, p<.05
		Stability in patient-provider relationships as measured by length of working relationship with providers (psychiatrist, case manager, and therapist)	Ware Continuity of Care Measure	Pyschiatrist at 9 months: p=.05<br>Case manager at 9 months: p<.05
		Stability in patient-provider relationship as measured by number of changes in providers (psychiatrist, case manager, and therapist)	Ware Continuity of Care Measure	Case manager/therapist at 18 months: p=.01
		Severity of instability in patient-provider relationship as measured by mean score of perceived quality of transition care by psychiatrists and case managers/therapists	Ware Continuity of Care Measure	Psychiatrist transition at 9 months: b=37.5, p=.03
		Hospitalization/Emergency Services: any psychiatric rehospitalizations	Personal History Form	OR=.11, 95% CI: .01-.96
		Family Support: mean score of frequency of family contact subscale	Lehman Quality of Life Interview	18 months: b=.47, p=.02
		Family Support: mean score of satisfaction with family relationship subscale	Lehman Quality of Life Interview	9 months: b=.66, p=.02
Lako et al., 2018	9 months	Mental Health: mean score of PTSD symptom scale	Impact of Event Scale	Mean difference= - 7.27, 95% CI= -14.31 - -.22, p=.04
		Mental Health: mean score of depression scale	Center for Epidemiological Studies	No effect
		Mental Health: mean score of Global Severity Index	Brief Symptom Inventory	No effect
		Mental Health: mean score of self-esteem scale	Rosenberg Self-Esteem Scale	No effect
		Familiy Support: mean score of availability of family support scale	Adapted from RAND Course of	No effect
		Social Support: mean score of availability of friends or other acquaintances scale	Adapted from RAND Course of	No effect
		Quality of Life: mean score of general life satisfaction defined as how the participant feels about their life in general	Lehman Quality of Life Interview	No effect
Samuels et al., 2015 (Shinn et al., 2015)	15 months	Housing: probability of remaining in homeless shelter	Residential Follow-Back Instrument	Significantly fewer days in the shelter until rehoused among FCTI
		Service Engagement: any mental health service use	Participant Interview	n/a
		Mental Health: mean Global Severity Index score	Brief Symptom Inventory	No effect
		Mental Health: mother-reported internalizing behaviors of children	Child Behavior Checklist	Ages 1.5-5 years: b=-3.65, p<.01
		Mental Health: mother-reported externalizing behaviors of children	Child Behavior Checklist	Ages 1.5-5 years: b=-3.12, p<.01
		Mental Health: child-reported internalizing behaviors	Youth Self-Report	No effect
		Mental Health: child-reported externalizing behaviors	Youth Self-Report	No effect
		Mental Health: child-reported depressive symptoms	Children's Depression Inventory	No effect
Shaw et al., 2017	6 weeks, 6 and 12 months	Service Engagement: proportion of participants still engaged with community mental health team	Participant Interview	6 weeks and 6 months: p<.05
		Service Engagement: engagement with mental health services (care coordinator, care plan, medication)	Participant Interview	6 weeks and 6 months: p<.05
Susser et al., 1997 (Herman et al., 2000; Jones et al., 2003)	18 months	Housing: number of homeless nights	Personal History Form	p=.003
		Housing: risk of homelessness	Personal History Form	Relative risk=.36, 95% CI=.12-1.06
		Housing: extended homelessness as measured by more than 54 nights	Personal History Form	Relative risk=.53, 95% CI=.27-1.01
		Housing: intermediate homelessness as measured by 30-54 nights	Personal History Form	No effect
		Housing: transient homelessness as measured by 1-29 nights	Personal History Form	No effect
		Mental Health: mean positive symptoms scale	Positive and Negative Syndrome Scale	No effect
		Mental Health: mean negative symptoms scale	PANSS	p=.02

		Mental Health: mean general psychopathology scale	PANSS	No effect
Stergiopoulos et al., 2017	12 months	Service Engagement: visits to primary care providers	Administrative data	No effect
		Hospitalization/Emergency Services: length of stay (days) in the hospital	Administrative data	No effect
		Hospitalization/Emergency Services: frequency of hospital admissions	Administrative data	No effect
		Hospitalization/Emergency Services: frequency of emergency department visits	Administrative data	No effect
		Mental Health: mental health severity in the past month	Colorado Symptom Index	No effect
		Substance Use: alcohol and drug addiction severity	Addiction Severity Index	No effect
		Quality of Life: health-related quality of life as measured by the means physical health and mental component subscale scores	Symptom Functioning-12	No effect
		Quality of Life: overall health quality of life	EuroQoL- 5 Dimension Visual Analogue	No effect
		Quality of Life: disease-specific quality of life	QoLI-20	Mean difference=-9.12, 95% CI=-17.57- -.67, p=.04
**Quasi-Experimental**				
Kasprow & Rosenheck, 2007	12 months	Housing: number of days housed (i.e., living in own home or with others)	Self-report Interview	p<.01
		Housing: number of days in institution (i.e., hospital, residential treatment, or jail)	Administrative data	p<.05
		Housing: number of days homeless	Self-report Interview	No effect
		Mental Health: level of psychiatric symptoms	Addiction Severity Index	p<.001
		Substance Use: level of alcohol use	Addiction Severity Index	p<.001
		Substance Use: level of drug use	Addiction Severity Index	p<.01
		Substance Use: alcohol use expenditures	Not specified	p<.05
		Substance Use: drug use expenditures	Not specified	p<.05
Nossel et al., 2016	12 months	Service Engagement: any outpatient service use	Medicaid Claims data	CTI reportedly had significantly greater contact with outpatient services.
		Hospitalization/Emergency Services: any emergency or inpatient service use	Medicaid Claims data	Reduction by .46 times for both groups, 95% CI=.35-.61
Shaffer et al., 2015	3 months	Service Engagement: substance use disorder outpatient service use	Administrative Claims data	No effect
		Hospitalization/Emergency Services: early hospital readmission within 30 days	Administrative Claims data	OR=2.83, 95% CI=1.72-4.65, p<.001
		Hospitalization/Emergency Services: long-term hospital readmission within 31-180 days	Administrative Claims data	No effect
		Hospitalization/Emergency Services: mental health crisis service use	Administrative Claims data	No effect
		Hospitalization/Emergency Services: mental health outpatient service use	Administrative Claims data	No effect
